# Traditional healing in Kisii County, Kenya: a personal narrative

**DOI:** 10.1192/bji.2023.15

**Published:** 2023-11

**Authors:** Lydia Matoke

**Affiliations:** Traditional Healer, Kisii County, Kenya. Email: lkmatoke@yahoo.com

**Keywords:** Traditional healers, healing, medicine, Kisii beliefs, herbology

## Abstract

This article describes the author's experiences growing up in a family of traditional healers, an account of early guidance by her grandmother, a severe illness that influenced her to become a healer and the values that are central to her own work as a traditional healer who specialises in treatment of mental health problems. The impact of colonisation on traditional healing practices in Africa is highlighted.

My name is Lydia Kemunto Matoke. I am a traditional healer from Kisii County in Kenya, about 300 km west of Nairobi. I am also a medical missionary in my church, a mother and grandmother. At the time of writing, I was the President of the Herbalists Society of Kenya. After 2000, as well as working as a healer in my own clinic, I was part of a number of joint traditional healing and government initiatives to develop regulatory structures to govern our profession in Kenya. Healing work for people with mental health problems has formed a major part of my practice.

Traditional healing in different parts of Africa takes many forms. However, I will share my story as an example of my life and development as a herbalist and healer in this part of Kenya. I am part of the Kisii tribe and our family are Kisii speaking. I have traditional healers going back many generations on both sides of my family. My mother's father was a healer. He specialised in head surgery. Many years before the use of modern Western anaesthetic and surgical techniques, my ancestors had access to surgical interventions with the aid of herbal preparations that provided pain relief, sedation and assisted with blood clotting and infection control.

## Colonial impact on traditional healing in Kenya

Prior to the colonisation of our country by Britain in the latter part of the 19th century, Indigenous healing knowledge was passed down from generation to generation in particular families, such as my family. With the arrival of the colonists, traditional approaches to health and healing were suppressed. Anything that the colonists did not understand was labelled as ‘witchcraft’. The religion they brought with them was used to justify the removal of Indigenous understandings and beliefs. In 1925 a law was passed in Kenya known as the Witchcraft Act, under which people accused of practising witchcraft were taken to court and could be imprisoned. Such legal practices resulted in the loss of Indigenous knowledge. Traditional healers became afraid to practise their healing and the general public became afraid to seek their help.

## Early experiences

My grandmother on my father's side lived in a different village many miles away but she was very influential for me. She specialised in traditional healing approaches for women with gynaecological problems. She would travel to visit me during my primary school years and take me out to pick herbs. My grandmother would hold my hand and walk with me, telling me the names of many plants and how to identify them. She would point out which herbs were helpful for which problems, for example those that were effective for infertility. She taught me which tools to use to gather each herb, the best methods for drying them and how to pound them into a powder with a wooden mortar. She explained which herbs were best boiled into a concoction and which were better dispensed as a powder. My grandmother often invited me to sit in with her when people came to our house to consult her. Patients would come and she would talk to them and ask questions. I observed the advice that she gave.

She taught me that food prepared in the right way is medicine. She recommended particular green leafy vegetables to boost immunity. She counselled against using metal utensils, prefering containers and pots made from clay to maintain the mineral and vitamin content of food. She explained that these practices had been handed down in our tribe over many generations. She cautioned against overeating as she said that would cause indigestion.

In my upbringing, I was exposed to traditional knowledge as well as Christianity. Even though my grandmother never learned to read or write and therefore couldn't read the Bible, she attended church. She was very clear that as a healer you have to lead a clean life in order to maintain the power of your healing. She encouraged me to tell the truth. She told me it was important not to get mixed up with crooked people. She was clear that it was important not to exploit people. She also had strong values around protecting patients’ privacy and told me all traditional healers have a special consultation room, whether that be a small hut in the compound or an extension of the main house.

My grandmother considered her healing art to be a wonderful heritage from God. She was concerned that we were in danger of losing these old traditions. She emphasised to me the importance of valuing Kisii culture and how our traditions have evolved over many generations to best meet our healing needs. Although she recognised the benefit of Western education and encouraged me in my school studies, she reminded me not to throw away our Indigenous knowledge. This was knowledge she was born with. She taught me the importance of dreams and visions in guiding her healing work. Sometimes she would wake in the morning and recall a dream in which she had been shown a herb necessary for healing a particular person. Other times a vision while awake revealed to her the best treatment for another person. Sometimes she heard voices in dreams or while awake advising her about particular treatments. Some traditional healers rely on ancestral spirits to guide their work, but my grandmother explained to me that God would speak to those who were healing others.

My grandmother was widely known in her area. Like other traditional healers she never had to advertise her work. People who had a good experience of her healing work would tell others and her reputation developed by word of mouth.

When I went to high school, my grandmother travelled a long distance from her home to meet with my school principal. She told her that she was giving me to them to educate, but they needed to understand that I would return to her one day as I was destined to become a traditional healer. In our tribe, such knowledge is always passed down to the most appropriate grandchild. My grandmother explained to me that she had been told by ancestral spirits to choose me.

## Illness

After high school, I had a number of jobs, completed a finance qualification and ended up in banking. However, when I was 27 years old, I was diagnosed with breast cancer. This was a very difficult time for me. My youngest daughter was only 4 years old. I underwent surgery to remove the cancer and then received several courses of chemotherapy and radiotherapy. However, by my early 30s, the cancer returned with metastases. Eventually my treating doctor told me there was no more treatment that could help, and advised me to return home. I was given medical retirement from my job.

I was determined not to give up because of my young daughter and family. During this time, I turned to my faith and changed my lifestyle. I sought advice from natural health practitioners and changed my diet to maximise green leafy vegetables that would boost my immunity. I consulted a herbalist and was prescribed treatments to address the cancer. It took a further 2 years for me to recover from the cancer. This experience had a strong influence on my faith in herbal treatments.

## Further training

It was soon after this time, when I was 33 years old, that my grandmother called me to see her. She was very old at that time and told me it was time to pass on her healing knowledge to me. She asked me to take on this healing in our family and community. This request created a big dilemma for me. On the one hand I respected her wish. On the other, as a Christian, I knew that the church did not accept traditional Kisii beliefs and values. As a result, I prayed about it, fasted and after some time, God gave me an insight that guided my decision. I realised that God created humankind from the earth and that Kisii culture is part of who we are and part of God's creation. I decided to continue the tradition handed down to me by my grandmother. As well as my grandmother's informal education I also studied in more formal settings. Many patients come to traditional healers with mental health problems. So I studied mental health and psychological counselling. Later, I completed a PhD in Herbology and Phytotherapy. However, none of those Western educational experiences gave me the practical grounding and spiritual insight that I received from my grandmother.

My grandmother taught me that when I do healing work, any cure does not come from me. Instead, it is a blessing that has been bestowed on the patient by the Creator, by God. She instilled in me that love is the first point of healing. She told me that when a practitioner is full of love and compassion, the patient tends to open up and talk to them. Then they can gain a much better understanding of the problem. This is particularly so for mental health problems.

## Recent developments in Kenya

In the past 20 years, the Herbalists Society of Kenya has participated with the Ministry of Health in working towards government policies to help regulate our sector. I have developed a College of Traditional Medicine so that traditional healing skills can be taught as a more formal training programme, alongside the traditional ways that such knowledge has usually been handed down through the generations. We are working towards documentation of such practices, to foster mutual understanding with Western medical practitioners. We have participated in mental health research.

## Conclusions

This paper describes experiences in my life that have shaped my values and work as a traditional healer in Kisii County in Kenya.

## Data Availability

Data availability is not applicable to this article as no new data were created or analysed in this study.

